# Supplementary material for: What We Know So Far about the Metabolite-Mediated Microbiota-Intestinal Immunity Dialogue and How to Hear the Sound of This Crosstalk
Source: Metabolites. 2021 Jun 21;11(6):406. doi: 10.3390/metabo11060406 (PMC8234899; doi:10.3390/metabo11060406)
Supplement: Supplementary file 1 [file metabolites-11-00406-s001.zip › metabolites-1244530-supplementary.pdf]

Table S1: Summarizing table of Microbiota-derived metabolites that modulate host immunity in the gut.

Metabolite:		Major Effects:	Ref:
**SCFAs**		• Maintain intestinal barrier integrity<br>• Induce of tolerogenic DC<br>• Butyrate promotes regulatory T cells and IL22 production	[28,29,31,32,35–37]
Lactate		• Suppresses the inflammation through the activation of GPR81 on DCs and macrophages<br>• Promotes M2 polarization<br>• Inhibits the activation of IECs<br>• Decreases the proliferation of effector T cells and increases the conversion of naive T cells to Tregs	[52,53,54,56]
Succinate		• Activates the SUCNR1 receptor and enhances its synthesis on macrophages<br>• Potent activator of tuft cells and leads to the activation of ILC2s	[61,62,66–68]
B Vitamins	Thiamine	• Required in lymphocyte survival	[86]
	Riboflavin	• Decreases TNF-alpha production by macrophages<br>• Enhances ROS production and phagocytic activity of macrophages<br>• Lessens clinical symptoms and systemic inflammation in patients with Crohn's disease	[90,91,95,96]
	Niacin	• Regulates ROS production by macrophages and neutrophils<br>• Enhances neutrophils recruitment and activity<br>• Promotes T-helper activation	[103–105]
	Pantothenic acid	• Enhances cytokines production and bacterial clearance in *M. tuberculosis* infection	[108]
	Pyridoxine	• Polarizes T-helpers towards a Th1 profile	[122–124]
	Biotin	• Correlates to histological gut inflammation in IBD-mice<br>• Enhances IL-17 and INF-γ production in T lymphocytes	[128,129]
	Folate	• Blood levels correlate to asthma severity and circulating lymphocyte levels in allergic children	[142]
	Cyanocobalamin	• Blood levels correlate to severity of infectious diseases in hospitalized patients	[138,139]
Tryptophan- derived indoles		• Increase the expression of IL10R1 in epithelial cells<br>• Increase the production of IL-22 in IELs<br>• Increase the number of ILC3 producing IL-22	[154,158,160]
Taurine		• Increases the number of lymphocytes in Peyer's patches<br>• Activator of the NLRP6 inflammasome and induces the production of AMPs	[168]
p-Cresol		• Inhibits the proliferation of CD43+ B-cell progenitor<br>• Increases ROS and Phagocytosis in macrophages<br>• Suppresses IFN-γ-producing Th1 cells and favors a Th2 response	[174,175,176]

Histidine	• It is a cytokine and inflammatory mediator in acute inflammation and hypersensitivity<br>• Promotes Th1 response through H1R while H2R activation induces polarization into regulatory T cells<br>• Diminution of IL-18 production through inhibition of NLRP6 inflammasome assembly	[169,179,183–185]
Spermine	• Reduces the expression of pro-inflammatory cytokines produced by monocytes and macrophages	[193,194]
Spermidine	• Reduces monocyte-derived pro-inflammatory cytokines production<br>• Controls the differentiation of naïve T cell to FoxP3+ T cells.	[201,202]
D-amino acids	• Metabolized by the epithelium to generate H_2O_2	
GABA	• Increases the production of IL-17<br>• Stimulates mucin-1 expression in epithelial cells<br>• Decreases IL-1β, increases tight junction and TGF-β in epithelial cells	[213,214,215]
QSMs	• Elicit proinflammatory effects and modulate the activities of gut-associated T lymphocytes, macrophages, DCs, and neutrophils<br>• Induce Mast cells degranulation and release of ROS, TNF- , and Prostaglandin D2	[221,222]
Catecholamines	• Act as potential inter-kingdom signaling molecules in the gut<br>• Exert autoregulatory functions on immune cells by means of intracellular oxidative mechanisms	[234,245]
CDNs and CTNs	• Induce either STING or RECON and induces antibacterial activity	[249,250,251]
Inosine	• Activates the adenosine 2A receptor and drive Th1 differentiation	[252]
Secondary Bile acids	• Negative regulator of macrophages, DC and NK<br>• Maintain a tolerogenic environment in the liver and in the intestine<br>• Promote generation of colonic RORγt-expressing FoxP3+ Tregs	[255,256,257,258,260]
Sphingolipids	• Preserve the integrity of the intestinal barrier<br>• Bacteroides-derived sphingolipids have anti-inflammatory properties<br>• Prevent accumulation of colonic iNKT	[266,271,270]
Lipoteichoic acids	• Protecting effect on intestinal mucosa<br>• Immunostimulatory properties through TLR2 on monocytes and macrophages<br>• Involve in the antibacterial immune response	[277,279,280,281–283,285,287]
